# Paroxysmal Finger Hematoma—A Probable Vascular Disorder in Post-COVID-19 Condition: Two Clinical Case Presentations

**DOI:** 10.3390/medicina58070915

**Published:** 2022-07-10

**Authors:** Hristo Abrashev, Julian Ananiev, Ekaterina Georgieva

**Affiliations:** 1 Department of Special Surgery, Orthopedics, and Traumatology, Clinic of Vascular Surgery, Medical Faculty, Trakia University, 6000 Stara Zagora, Bulgaria; hristo.abrashev@trakia-uni.bg; 2 Department of General and Clinical Pathology, Forensic Medicine and Deontology and Dermatovenereology, Medical Faculty, Trakia University, 6000 Stara Zagora, Bulgaria; julian.r.ananiev@trakia-uni.bg; 3 Department of Chemistry and Biochemistry, Medical Faculty, Trakia University, 6000 Stara Zagora, Bulgaria

**Keywords:** Achenbach’s syndrome, post-COVID-19, endothelial dysfunction, paroxysmal finger hematoma, vascular disorders

## Abstract

*Background and Objectives*: Achenbach’s syndrome is usually a benign, self-limiting clinical condition presented with finger discoloration, pain, and edema. Etiology, pathogenesis, and incidence remain unknown due to the variety of clinical features and the diversity of disease states leading to digital ischemia. COVID-19 primarily affects microcirculation, causing endothelial damage and disseminated microthrombosis. *Materials and Methods*: We reviewed two cases of Caucasian women with Achenbach’s syndrome after COVID-19 infection recovery between April and May 2021. *Results:* Here are presented two extremely rare cases of paroxysmal finger hematoma in two female patients after COVID-19 infection recovery. *Conclusions:* The exact etiology and pathophysiology of Achenbach’s syndrome remain unclear. It is assumed that SARS-CoV-2 infection could be the triggering factor in the pathophysiological mechanism of paroxysmal finger hematoma. We highly recommend the implication of the synthetic prostacyclin receptor agonist (Iloprost) as a first-line conservative treatment in patients with Achenbach’s syndrome and COVID-19 infection recovery.

## 1. Introduction

Achenbach’s syndrome (AS), also known as “painful blue finger” or “paroxysmal finger hematoma”, is a rare clinical condition that results in the sudden onset of bruising along with burning pain and swelling in one or more digits [1], with the subsequent appearance of a hematoma on the volar aspect of the proximal phalanges without a history of prior trauma [2,3]. Its clinical presentation may mimic acute limb ischemia, Raynaud’s, Buerger’s disease, and other medical conditions, but normally the evident subdermal bleeding are self-limiting and have a spontaneous resolution. Achenbach’s syndrome is 2–7 times more common in middle-aged women than in men, and Caucasians are more affected [4]. Although the syndrome is usually presented as a benign condition, it can cause serious unrest for patients. However, the etiology of the “painful blue finger” is still unknown, but there are a few studies that speculate on its etiopathogenesis [5]. Typically, symptoms resolve without medical or surgical intervention. This study presents two female patients with paroxysmal finger hematoma after COVID-19 infection.

## 2. Materials and Methods

### 2.1. Case Report One

**Figure 1:**
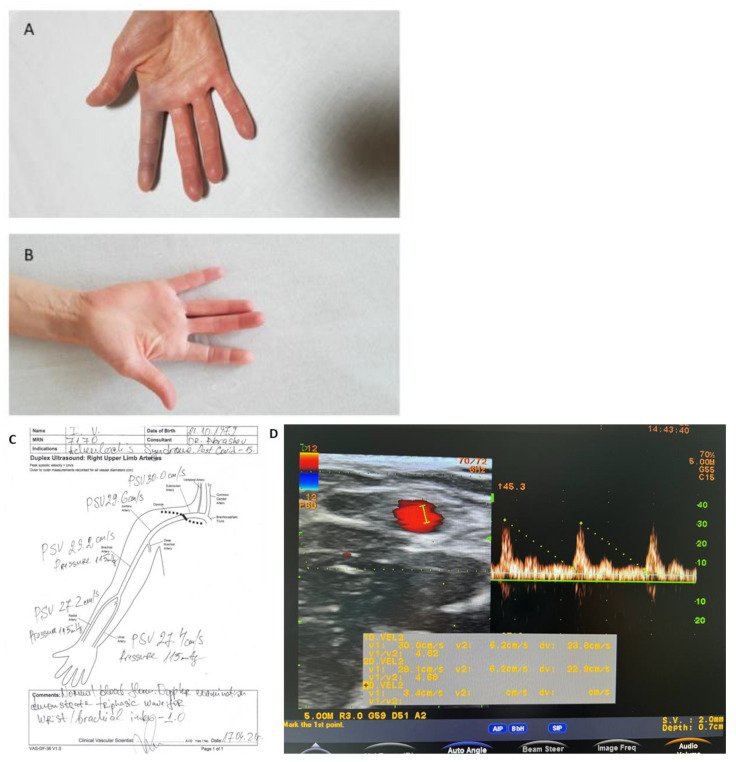
(**A**,**B**). A 43-year-old woman presented with AS symptoms including sudden pain, bruising, and edema on her right index finger. Hand pictures: (**A**) the first day on admission; and (**B**) the first day on discharge; (**C**,**D**). Data of Doppler investigation (**C**) and Doppler ultrasound result on the first day (**D**) showed normal peak forward velocities in cm/s. Doppler examination demonstrates triphasic waveforms. Brachial/wrist index of 1.0.

A previously healthy 43-year-old right-handed, non-smoking woman presented with spontaneous acute pain, swelling, paresthesia, and blue discoloration along the volar aspect of her right index finger, engaging all three phalanges. The patient also presented with extreme pain upon palpation and had restricted and painful ranges of motion (both passive and active) of the affected digit, whilst the Allen and Adson tests were normal. The symptoms began as a sudden pain with mild bruising and a loss of superficial sensation in the affected digit. Within 2 h of the onset, the index finger became unbearably painful and swollen, for which she was admitted to the vascular department. During a physical examination, the right index finger demonstrated fusiform swelling with notable bruising involving the entire finger (Figure 1A). 

Pulsations on both the right ulnar and radial arteries were normal and symmetrical. The sensitivity, skin color, local temperature, and active movements of the unaffected digits were preserved, as was that of the right wrist and forearm. Twenty days before the patient was admitted, she recovered from a mild clinical presentation of COVID-19 infection with no complications and received treatment with short-term antiplatelet therapy. Neither previous trauma nor cold exposures were reported, and she had an unremarkable medical history without associated diseases. All blood tests, including a complete blood count, rheumatologic screening, autoantibody panel tests, coagulation tests, coagulation factors tests, and inflammation markers, were normal (Table 1). Doppler ultrasonography showed normal blood flow in the arteries on both hands: subclavian, brachial, radial, ulnar, and digital arteries of the affected hand (Figure 1D). However, the finger plethysmography revealed a slight difference between the pressures of an affected finger and those of the unaffected hand: 117 mmHg versus 114 mmHg in the index finger of the unaffected hand. The chest X-ray was normal with no sign of post-COVID-19 infection sequelae.

### 2.2. Case Report Two

**Figure 2:**
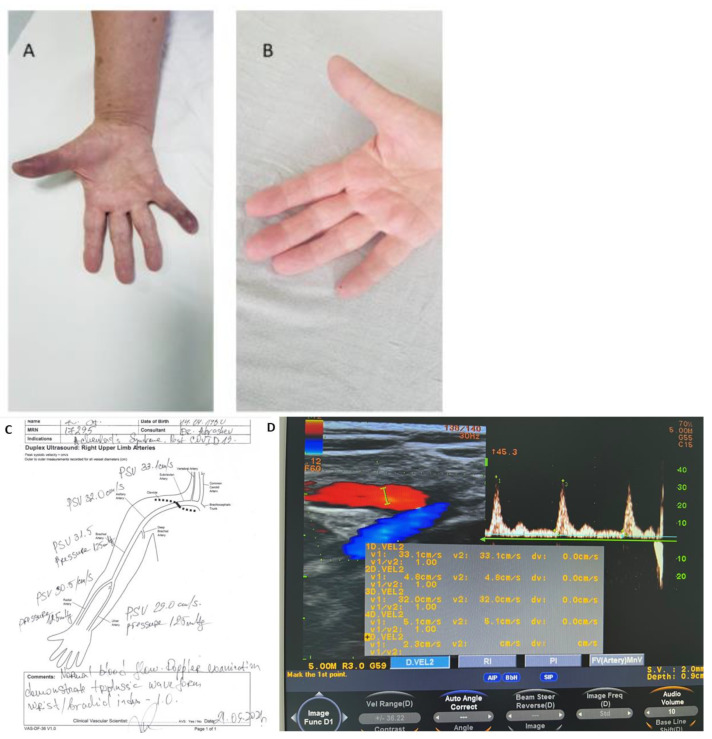
(**A**,**B)**. A 71-year-old non-smoking woman presented with sudden swelling and bruising along the volar aspect of her first and fifth fingers on her right hand. Hand pictures: (**A**) the first day of admission; and (**B**) the first day of discharge. (**C**,**D**). Data of Doppler investigation (**C**) and Doppler ultrasound result (**D**) showed normal peak forward velocities in cm/s. Doppler examination demonstrates triphasic waveforms. Brachial/wrist index of 1.0.

The second patient was a 71-year-old right-handed, non-smoking woman, who presented with sudden onset of pain, swelling, bruising along the volar aspect of her first and fifth fingers on her right hand, and loss of sensation. There was no history of causative triggers such as preceding trauma, cold exposure, stress, medical intoxication, and no previous similar episodes (Figure 2A). 

On physical examination, she had a semi-restricted range of motion with mild pain to palpation, diminished local temperature with partial loss of sensation, and blue discoloration of the affected digits. Similar to the first patient, pulsations on both right ulnar and radial arteries were normal and symmetrical. The sensitivity, skin color, local temperature, and active movements of the unaffected digits were preserved. Doppler ultrasonography showed a normal blood flow in the arteries on both hands: subclavian, brachial, radial, ulnar, and digital arteries of the affected hand (Figure 2D). Plethysmography showed no difference between the pressures of affected fingers. The Allen and Adson tests were normal.

The patient stated that 4 months ago she had a mild to severe presentation of the COVID-19 infection for which she was hospitalized. The patient’s clinical history involved thyroid cancer (T2N × M0) and a subsequent thyroidectomy 7 years ago. Routine blood screening tests were performed and the following changes were found: elevated ASAT (148.8 U/I), ALAT (138.4 U/I), LDH (987.0 U/I), and CRP (11.5 mg/L). Doppler ultrasonography of the arteries of the upper extremities was normal. The finger plethysmography showed no significant difference (affected finger 117 mmHg) compared to the other digits (mean range 116 mmHg). A typical post-COVID-19 infection change with multifocal reticular opacities bilaterally and pulmonary fibrosis was revealed. Worthy of note, the patient reported no pulmonary problems.

## 3. Results

The pathogenesis of coronavirus infection has been well researched and is related to a high incidence of thromboembolic phenomena [6] and endothelial damage [7,8,9]. In endothelial cells, the increased expression of angiotensin-converting receptors 2 (ACE2) can disseminate a cycle of endothelial inflammation and thromboembolic phenomena in post-SARS-CoV-2 infection [10]. According to this, recent recommendations for the usage of antithrombotic prophylaxis with anticoagulants such as heparin have been published [11]. Iloprost can restore normal endothelium function and normalize capillary resistance. For patients with a post-COVID-19 vascular complication, Faggioli and colleagues strongly recommend prostanoid infusion with a synthetic prostacyclin receptor agonist (Iloprost) [12]. Iloprost inhibits platelet aggregation and activation of leukocytes [13], which leads to down-regulation of adhesion molecule expression and improves endothelial function [14]. The mechanism included imitation of endogenous prostacyclin-PGI2 and binding to a G-protein, localized in the vascular smooth muscle receptor, which leads to the activation of a cyclic adenosine monophosphate, vascular smooth muscle relaxation [12]. Also, prostacyclin alleviates coagulopathy by controlling platelet aggregation through elevated nitric oxide production, which promotes vasodilatation and prevents vasoconstriction and platelet aggregation in cases of SARS-CoV-2-infection [15]. The safety, high-patient tolerability, and effective profile characterize Iloprost as a therapeutic agent with high applicability for treatment and recovery in patients with COVID-19 and AS.

The first patient was conservatively treated for 14 days and used as follows: intravenous infusion of 750 UI/h of Heparin for a day, followed by Nadroparin (Fraxiparine) 0.4 mL twice a day s.c (for 9 days), intravenous infusion of Iloprost Trometamol (Ilomedin) 20 ng/1 mL once a day (for 7 days), Papaverine hydrochloride 20 mg/1 mL twice a day (for 3 days), Clopidogrel 75 mg once a day (for 4 days) p.o and analgesia (Figure 1). On the 12th day, the patient reported pain and tension in the affected finger whilst the bruising was still present. On the 12th day, the patient reported pain and tension in the affected finger while the bruising was still present. On the 14th day, she was discharged with no pain and swelling and a decrease in bruising. Two days later, she was discharged with no pain or swelling and a decrease in bruising. Ambulatory therapy was prescribed for a month and consisted of Clopidogrel 75 mg once a day, Naftidrofuryl oxalate (Dusodril) 100 twice a day, and Calcium besylate (Doxium) 500 mg once a day. At the 12-week follow-up, the patient was asymptomatic and had fully recovered with no recurrence.

The conservative treatment for the second patient included continuous intravenous infusion of Heparin 750 UI/h for a day, followed by Nadroparin (Fraxiparine) 0.4 twice a day s.c, (for 4 days), Iloprost trometamol (Ilomedin) 20 ng/1 mL once a day (for 5 days), Papaverine hydrochloride 20 mg/1 mL twice a day (for 3 days), and analgesia. The patient’s condition gradually improved, and her symptoms had completely resolved within 5 days. She was subsequently discharged and had no complaints. Follow-up treatment included antiplatelet therapy with Clopidogrel 75 mg once a day, Naftidrofuryl oxalate (Dusodril) 100 mg twice a day, and Calcium dobesilate (Doxium) 500 mg once a day for one month (Figure 2). We are currently unable to monitor the patient’s recovery. It has not sought medical attention and has not been identified with further episodes.

## 4. Discussion

Often, paroxysmal finger hematoma is an underrecognized vascular syndrome with no epidemiological description to date and it predominantly affects the female gender [16]. A French study indicated that the condition is actually more common, with a prevalence of 12.4% in women and 1.2% in men, and the age of onset is predominantly over 50 years in the general population [1].

Plethysmography is a method that registers variations in blood volume or blood flow in the body and significant differences from plethysmography results are a hallmark of widespread small-vessel occlusion. Often, plethysmography results are abnormal even in the absence of obvious upper extremity symptoms or physical findings and are seen in patients with atherosclerosis, long-standing diabetes, chronic kidney failure, or systemic connective tissue diseases [17]. The results from finger plethysmography of the affected finger of the first patient show a slight deviation. As the first patient has a clear medical history (Figure 1), hypothetically, the difference in plethysmography results might be due to a late post-COVID complication or idiopathic. As the post-COVID condition is difficult to predict how long it will last after discharge, conservative treatment was prescribed for both patients [18]. While the first patient was asymptomatic and had fully recovered with no recurrence at the 12-week follow-up, the second patient was unfortunately not monitored and no data was available regarding her recovery.

While the etiopathogenesis of AS is unclear, some studies support the hypothesis of a vasomotor disorder. It is assumed that the intimate mechanism involves a vasomotor disorder, reduced capillary resistance, subcutaneous bruising (due to venous blood infiltration in the soft tissues), and diminished digital blood flow [19]. COVID-19 infection affects the vascular system, both macro, and microcirculation, therefore coronavirus infection could be considered a predisposing factor for developing AS. Whilst there is great data concerning the pathophysiology and the impairment of large vessel occlusions (e.g., acute stroke, coronary occlusion, pulmonary embolism, deep venous thrombosis), the consequences of microcirculatory disturbances remain unclear [20,21]. It has been found that the virus has the ability to connect to ACE 2 receptors on cell surfaces and enter the cells in most organs. Changes in cell morphology and apoptosis of endothelial cells are associated with COVID-19 infection. Several studies show that the infection generates hemorrhagic microscopic lesions in the brain as well as the skin and is associated with endothelial swelling and thrombosis, followed by fibrinoid necrosis of the surrounding tissue in some patients [22]. Furthermore, endothelial damage is likely to disrupt capillary flow patterns when erythrocyte diameters exceed capillary lumen diameters [23]. Regarding the second patient (Figure 2), besides the hypothetic COVID-19-related endothelium damage in the digit vessels, another study showed recurrent episodes of paroxysmal finger hematoma associated with hypothyroidism [24]. Venous thromboembolism prevention is an important component of the complex and comprehensive treatment of COVID-19 and post-COVID syndrome. As the common condition of both patients may rapidly deteriorate and result in dynamic modifications of the cardiovascular system with an increased risk of thrombosis, the administration of low molecular heparin and the antiplatelet drug was needed. Nadroparine is a classical first-line therapeutic anticoagulant and it is used for the treatment of COVID-19-related vascular complications [25]. Clopidogrel is a potent antithrombotic drug that inhibits platelet aggregation by inhibiting the adenosine diphosphate P2Y12 receptor. This indicates possible positive effects of Clopidogrel in patients with COVID-19 infection, preventing the onset of further cardiovascular adverse events [26].

Finally, the role of free radicals (ROS and RNS) in damaging the endothelium is well studied in severe infectious diseases, including COVID-19 [27,28,29]. Free-radical-induced endothelial dysfunction in viral infections triggers coagulation disorders and can be caused directly by the viral attack [30]. Montiel and colleagues report that SARS-CoV-2 leads to inflammation, decreased nitric oxide (NO) bioavailability, and an imbalance of NO/ROS, which causes endothelial injury, hypercoagulable state, and microangiopathy [31]. These pathophysiological mechanisms could be a triggering factor for reduced capillary resistance, microvascular fragility, subdermal bleeding, diminished digital blood flow, and hypoxia [32]. Clinical trials suggest that endothelial dysfunction is a hallmark of coronavirus disease and infection pathology is characterized by oxidative stress [30] and cytokine storm, which results in endothelial inflammation [33,34]. 

## 5. Conclusions

Achenbach’s syndrome as a result of long-COVID-19 has not been reported in medical literature, making it both interesting and demanding to diagnose and treat. Since coronavirus infection plays an important role in the pathophysiology mechanisms initiating endothelial dysfunction, we suppose that AS without a genetic predisposition can be classified as a specific condition, attributed to post-COVID-19. As both AS and COVID-19 infection effects have moderate and alternate microcirculatory circulation, treatment in such cases should first prioritize the prevention of potentially life-threatening conditions. Further studies need to be carried out to clarify the role of coronavirus infection in the initiation and progression of AS.

## Figures and Tables

**Table 1:** Standard blood screening test results of 43-year-old woman. The data does not show deviations from the reference ranges.

Test	Result	Unit	Reference Range
Leukocytes, (WBC)	6.9	g/L	3.5–10.5
Lymphocytes, (LYM)	30.3	%	20–48
Neutrophils, (Neu)	61.9	%	40–70
Monocytes, (Mo)	7.8	%	1–11
Erythrocytes, (RBC)	4.56	×10^12^/L	3.7–5.3
Hemoglobin, (HGB)	139	g/L	120–160
Hematocrit, (HCT)	0.392	L/L	0.360–0.480
Mean corpuscular volume, (MCV)	86.1	fL	80.0–96.0
Mean corpuscular hemoglobin, (MCH)	30.4	pg	27.0–33.0
Red Cell Distribution Width, (RDW)	15.1	%	11.2–14.7
Platelet Count, (PLT)	239	×10^9^/L	130–140
Mean Platelet Volume, (MPV)	7.3	fL	6.3–12,5
Glucose	5.64	mmol/L	3.6–6.1
Creatinine	60	µmol/l	65–127
Uric acid	201	μmol/L	142–340
Erythrocyte Sedimentation Rate, (ESR)	5	mm/h	0–39
Total Bilirubin	6.1	µmol/L	<21
Total protein, (TP)	78.5	g/L	64.00–83.00
Albumin	47.1	g/L	35.00–52.00
Aspartate Aminotransferase, (ASAT)	20.4	U/L	<40.00
Alanine Aminotransferase, (ALAT)	17.3	U/L	<33.00
Creatine Kinase, (CK)	88	U/l	<170.0
C-reactive protein, (CRP)	0.2	mg/l	<5
Factor V Leiden, (FVL)	Negative	PCR-RFLP	
Sodium levels, Na+	136	mmol/L	136.00–151.00
Potassium levels, K+	4.1	mmol/L	3.50–5.60
Rheumatoid Factor (RF)	8	IU/mL	<20
Fibrinogen	2.12	g/L	2–4 g/L
International Normalized Ratio (INR)	0.94	UI	0.8–1.2
Prothrombin time (PT)	108.9	%	70–130
Activated Partial Thromboplastin Time (APTT)	31.1	sec	27.6–37.2

## Data Availability

The personal data presented in this study are not publicly available due to privacy restrictions. The data are available on request from the corresponding author.
